# Development of SNP-LAMP Combined with Lateral Flow Dipstick to Detect the S531L *rpoB* Gene Mutation in Rifampicin-Resistant *Mycobacterium tuberculosis*

**DOI:** 10.3390/diagnostics15172183

**Published:** 2025-08-28

**Authors:** Jutturong Ckumdee, Monpat Chamnanphom, Supaporn Wiwattanakul, Somchai Santiwatanakul, Kwanchai Onruang, Thongchai Kaewphinit

**Affiliations:** 1 Department of Clinical Pathology, Faculty of Medicine, Vajira Hospital, Navamindradhiraj University, Bangkok 10300, Thailand; jutturong@nmu.ac.th; 2 Center of Excellence in Medical and Environmental Innovation Research (CEMEIR), Srinakharinwirot University, Bangkok 10110, Thailand; 3 Department of Pathology, Faculty of Medicine, Srinakharinwirot University, Nakhon Nayok 26120, Thailand; monpat@g.swu.ac.th (M.C.); titi41@yahoo.com (S.S.); kwanchai@g.swu.ac.th (K.O.); 4 Innovative Learning Center, Srinakharinwirot University, Bangkok 10110, Thailand

**Keywords:** *Mycobacterium tuberculosis*, MDR-TB, rifampicin resistance, LAMP, S531L mutation, lateral flow dipstick, rapid tuberculosis diagnosis

## Abstract

**Background:** Tuberculosis (TB) remains a primary global health concern, despite the widespread availability of effective chemotherapeutic interventions. The emergence and dissemination of drug-resistant strains of *Mycobacterium tuberculosis*, particularly those exhibiting resistance to rifampicin, present significant obstacles to the success of TB control programs. Consequently, there is an urgent need for rapid, sensitive, and specific molecular diagnostic tools to inform timely clinical decision-making and reduce the transmission of disease. Loop-mediated isothermal amplification (LAMP) has gained attention as a promising alternative to conventional polymerase chain reaction (PCR) techniques. This method, which facilitates DNA amplification under constant temperature conditions, offers advantages including high specificity, rapid turnaround time, and operational simplicity—features that render it especially suitable for implementation in resource-limited settings. **Methods:** In this study, a LAMP assay targeting the *rpoB* gene was developed, with particular focus on detecting the codon 531 C→T mutation associated with rifampicin resistance. A set of four to six primers was designed to recognize six distinct regions of the target sequence. Allele-specific amplification was achieved by incorporating a deliberate single nucleotide mismatch at the 3′ terminus of the B2 primer to enable precise discrimination between wild-type and mutant alleles. The assay was conducted at an optimized temperature of 61 °C for 60 min, followed by visual detection using a lateral flow dipstick (LFD) within five minutes. **Results:** The LAMP-LFD assay demonstrated 100% concordance with drug susceptibility testing (DST) and DNA sequencing. No cross-reactivity with wild-type strains was observed, underscoring the assay’s high specificity. **Conclusions:** This platform offers a robust, field-deployable solution for detecting the codon 531 C→T mutation associated with rifampicin resistance in low-resource settings.

## 1. Introduction

The significant burden of tuberculosis (TB) remains a formidable public health challenge, despite the availability of effective treatment regimens. In 2023, an estimated 400,000 new cases of rifampicin-resistant or multidrug-resistant tuberculosis (RR/MDR-TB) were reported globally. The urgent need to address the epidemic of opioid-related deaths is underscored by the fact that only 44% of affected patients receive evidence-based treatment [1]. According to the WHO’s 2022 estimates, Thailand’s TB incidence is 155 per 100,000, with RR/MDR-TB at 3.7 per 100,000 [2].

The emergence of resistance to rifampicin (RIF) and isoniazid (INH), the two most effective first-line anti-TB drugs, poses a significant obstacle to global TB control efforts. With a cure rate of only 68% for drug-resistant TB, accurate and timely diagnosis is critical for guiding appropriate therapeutic interventions and curbing disease transmission. Rifampicin resistance, a key indicator of MDR-TB, is closely linked to specific mutations in the 81-base pair rifampicin resistance-determining region (RRDR) of the *rpoB* gene, particularly at codons 531, 526, and 516 [3,4,5].

Early diagnosis and prompt initiation of treatment are essential for controlling TB transmission [6]. In light of the limitations of traditional diagnostic methods, molecular techniques have been developed for the rapid detection of TB and drug susceptibility testing. While PCR-based technologies can address some of the shortcomings of classical phenotypic methods, high testing costs remain a significant barrier in clinical research, surveillance, and public health [7,8,9,10].

One notable advancement is the Xpert MTB/RIF assay (Cepheid, Sunnyvale, CA, USA), a cartridge-based, fully automated real-time PCR system that simultaneously detects *M. tuberculosis* and rifampicin resistance. This system has received WHO approval for the preliminary screening of MDR-TB cases due to its rapid testing capabilities and user-friendly design [11]. However, the widespread implementation of Xpert MTB/RIF in low- and middle-income countries is hindered by reliance on a stable electricity supply, the need for routine maintenance, and the high costs of equipment and reagents. These factors significantly impede sustainability and scalability, especially in high TB burden nations like Thailand [11].

Given the high prevalence of drug-resistant TB in low- and middle-income countries, there is an urgent need for affordable, simple, and field-friendly diagnostic tools. As rifampicin remains the cornerstone of first-line TB treatment, resistance to this drug complicates care, increases costs, prolongs treatment duration, and diminishes patient response [12]. Over 95% of rifampicin-resistant strains harbor mutations in the *rpoB* gene RRDR, making this region a critical focus for diagnostics [6]. While rare mutations upstream of the RRDR have been identified, the majority of clinical rifampicin resistance is attributed to mutations within this essential hotspot.

In response to these challenges, there is an urgent need for rapid, cost-effective, and user-friendly diagnostic platforms that are disruptive and suitable for point-of-care testing (POCT) in underserved settings. Such tests could significantly enhance the early detection of MDR-TB, leading to improved patient outcomes in resource-limited environments with restricted access to centralized laboratory facilities.

The loop-mediated isothermal amplification (LAMP) assay offers enhanced sensitivity and specificity for the early diagnosis of drug resistance in *M. tuberculosis*. Recent improvements have enabled the accurate detection of single-nucleotide polymorphisms (SNPs) and short insertions/deletions (indels). Additionally, several methodological modifications have significantly improved its ability to distinguish between wild-type and mutant alleles with remarkable accuracy [13,14,15,16,17,18,19,20,21,22,23,24].

However, existing LAMP systems for SNP detection face technical limitations. Primer design is particularly challenging, as the placement of mismatches between the primer and allele sequence must be strategic to promote allele-specific amplification while minimizing off-target reactions. This non-standard process is challenging to optimize. Although the LFD system is user-friendly, it may yield false positives or negatives due to incomplete hybridization or nonspecific binding [25,26,27,28,29,30,31,32]. Furthermore, LAMP’s inherent high amplification efficiency poses risks of carry-over contamination and false results if rigorous laboratory protocols are not followed.

Against this backdrop, our study developed an LFD to detect the S531L [C→T] mutation in the *rpoB* gene, a known marker of rifampicin resistance in MDR-TB, utilizing a LAMP reaction. We employed a novel approach to primer design, introducing a 3′-end modification on the B2 primer to facilitate allele-specific amplification. This design preferentially amplified mutant alleles while effectively suppressing the amplification of wild-type DNA. The result readout was straightforward, and rapid visual detection was easily achieved due to the LFD format.

The newly developed LAMP-LFD assay demonstrated high sensitivity and specificity for detecting the S531L [C→T] substitution in the *rpoB* gene. It performed well in mixed samples of wild-type and mutant alleles, indicating its potential for POCT without reliance on expensive laboratory techniques. Moreover, this method represents a promising advancement in molecular diagnostics for detecting mutations that confer resistance to treatment. It can be easily adapted for identifying other clinically significant genetic alterations in *M. tuberculosis* and potentially other pathogens.

## 2. Materials and Methods

### 2.1. Ethical Approval

In this study, the genomic DNA of *M. tuberculosis* was isolated from previously cultured colonies obtained during routine diagnostic procedures. To ensure the protection of patient confidentiality, all DNA specimens were irreversibly anonymized, with no identifiers linking them to individual patient data, before researcher access and downstream utilization. The study protocol was fully approved by the Human Research Ethics Committee of Srinakharinwirot University (certificate of approval number SWEC/X-023/2566).

### 2.2. Mycobacterium Specimens

Ten clinical sputum specimens (six drug-resistant and four drug-susceptible), *M. tuberculosis* H37Rv, non-*M. tuberculosis* (NTM; *Mycobacterium avium* and *Mycobacterium intracellulare*), and RRDR 531 mutant reference strains were obtained from the National Tuberculosis Reference Laboratory, Thailand. DNA extraction involved sputum decontamination with *N*-acetyl-l-cysteine–sodium hydroxide, followed by centrifugation, resuspension in distilled water, heat inactivation at 95 °C for 20 min, and sonication for LAMP amplification. Sputum specimens were identified using culture-based comparison on Lowenstein–Jensen slants incubated at 37 °C for up to 8 weeks. Genomic DNA was prepared according to Kaewphinit et al.’s method [9]. All DNA samples received were initially tested using drug susceptibility testing (DST) to determine rifampicin resistance. Subsequently, the samples were analyzed using LAMP-LFD and DNA sequencing assays, comprising six drug-resistant and four drug-susceptible isolates.

### 2.3. Primer Design and LAMP Amplification

LAMP primers were designed based on published sequences of the *rpoB* gene, which encodes the RNA polymerase beta subunit and carries the S531L [C→T] mutation. The B2 primers were modified explicitly so that the nucleotides at positions 1 and 3 from the 3′-end are identical to the nucleic acid sequence downstream of the fragment containing the targeted genetic variant. Accordingly, the B2 region of the BIP (backward inner primer) matches not only the sequence within the B2 region itself but also the sequence following the site of the mutation, including potential insertions and/or deletions.

The B2 primer is linked via its 5′ end, preferably through a TTTT bridge to the 3′ end of the B1c primer, which is designed to be reverse-complement to the target DNA fragment (5′→3′ strand) and positioned between the 3′ end of the F2 primer and the 5′ end of the F1c primer. The remaining LAMP primers, F3, B3, FIP (forward inner primer), and optionally LB (loop backward) primers, were designed according to standard LAMP protocols, as recommended in the literature or using bioinformatics tools such as Primer Explorer (https://primerexplorer.eiken.co.jp/lampv5e/index.html (accessed on 21 August 2025)).

**Figure 1:**
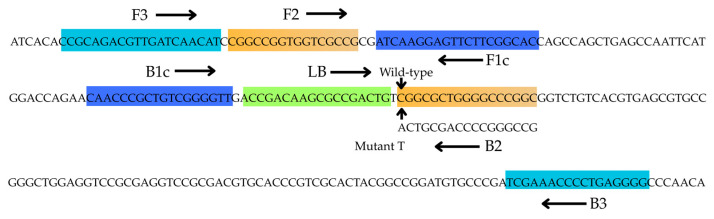
Nucleotide sequence of the *rpoB* gene [GenBank accession no. JX303320]. The primers F3, B3, FIP (F1c/TTTT/F2), BIP-biotin (B1c/TTTT/B2), and LB-fit-C-labeled probe were shown as nucleotide sequences and arrows.

This modified approach can also be applied to the F2 region of the FIP primer, in which case the FIP primer captures the mutation. As illustrated in Figure 1, the method developed in this study involves nucleotide modifications at positions 1 and 3 of the B2 region of the BIP primer.

These primer modifications confer high specificity to the mutated sequence, such that the amplification product is generated only in the presence of the mutant allele and not with wild-type genomic DNA. For primer mismatch optimization, DNA templates from *M. tuberculosis* strains harboring either the drug-sensitive or drug-resistant *rpoB* gene were used in LAMP reactions. The LAMP primers and LB-fit-C sequence are listed in Table 1.

LAMP primers for *M. tuberculosis* were based on the published sequence of the *rpoB* gene corresponding to an MDR-TB strain (GenBank accession no. JX303320). LAMP reactions were performed at temperatures below 61 °C for 1 h, followed by the analysis of amplification products via agarose gel electrophoresis.

Each 25 μL LAMP reaction mixture contained 2 µM each of inner primers FIP and BIP, 0.2 µM each of outer primers F3 and B3 and LB, 1.6 mM of dNTP mix (New England Biolabs Inc., Beverly, MA, USA), 0.5 M of betaine (Sigma-Aldrich, St. Louis, MO, USA), 6 mM of MgSO_4_, 8 U *Bst* WarmStart DNA polymerase (large fragment; New England Biolabs Inc., Beverly, MA, USA), 1× supplied reaction buffer, 1 μL of DNA template (5 ng), and 1 μL of distilled water for the negative control. All preparation steps were conducted on ice.

To prevent evaporation, 20 μL of mineral oil was added to each tube. The tubes were then incubated at 61 °C for 1 h in a water bath or heat block. The LAMP amplicons were terminated by heating at 95 °C for 2 min. Finally, 2% agarose gel electrophoresis was performed at 100 V to analyze the LAMP products, and the gel was visualized under UV light to confirm the amplification.

### 2.4. LFD Assay

**Figure 2:**
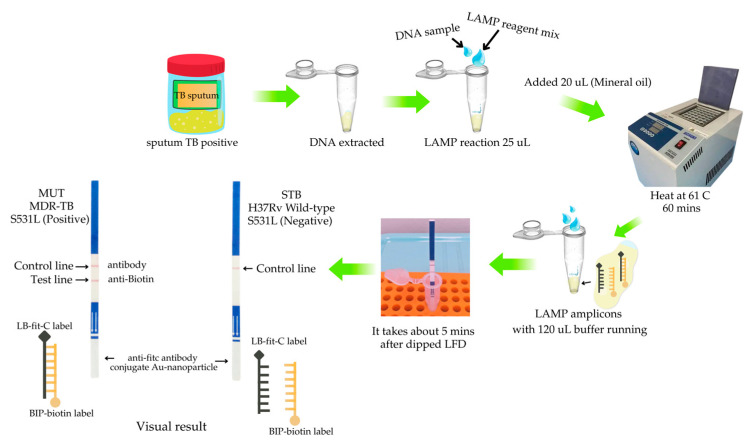
LAMP reactions were performed at temperatures below 61 °C for 1 h, followed by analysis of amplification products via the LAMP-LFD assay.

A 5′-fluorescein isothiocyanate (FITC)-labeled oligonucleotide probe was synthesized by Bio Basic Inc. (Markham, ON, Canada). This probe was specifically designed to hybridize with a segment of the *rpoB* gene previously identified as being associated with rifampicin resistance in *M. tuberculosis*. The target region is situated between the binding sites of the B1c and B2 primers. In accordance with the protocols established in prior diagnostic assays, 20 pmol of the FITC-labeled probe (designated LB-fit-C) was introduced at the initial stage of the reaction, thereby obviating the requirement for post-LAMP hybridization steps. Following amplification, the LAMP product was combined with 120 μL of assay buffer. Subsequently, a customized LFD, developed by K.BIO SCIENCES Co., Ltd. (Pathum Thani, Thailand), was inserted into the reaction mixture and incubated at ambient temperature for 5 min. The presence of hybridization signals was then visually detected by the unaided eye (Figure 2).

### 2.5. Sensitivity and Specificity of LAMP-LFD

The analytical sensitivity of the optimized LAMP-LFD assay was determined using a series of serial dilutions of genomic DNA extracted from bacterial strains previously characterized by drug susceptibility testing (DST). The DNA concentrations ranged from 50 nanograms to 5 femtograms (50 ng, 5 ng, 500 pg, 50 pg, 5 pg, 500 fg, 50 fg, and 5 fg). The tested samples included both wild-type and *rpoB* mutant strains, with the presence of the mutation at codon 531 confirmed through DNA sequencing. These DNA dilutions were subsequently employed to evaluate the specificity of the detection system. The specificity of the LAMP-LFD assay was verified by performing the LAMP reaction using DNA from the wild-type *M. tuberculosis* strain H37Rv, a rifampicin-resistant mutant, and non-*M. tuberculosis* (NTM). The assay was conducted and evaluated as described in the previous section. Each test was performed in triplicate, with three independent replicates.

### 2.6. Application of Sample Analysis

In this study, ten individual clinical sputum samples were used exclusively for the evaluation of the LAMP-LFD assay. The results obtained from the LFD assay were compared with those from drug susceptibility testing (Bureau of Tuberculosis, Bangkok, Thailand) and DNA sequencing tested by Sanger sequencing (Bio Basic Inc., Markham, ON, Canada) using F-*rpoB*: 5′-GCATGTCGCGGATGGAG-3′ and R-*rpoB*: 5′-CCCCTCAGGGGTTTCGA-3′, which covered a mutation position product of 299 bp.

## 3. Results

LAMP primers were designed based on point mutations (C→T) at codon 531 of the *rpoB* gene, which encodes the RNA polymerase β-subunit and is associated with rifampicin-resistant mutants (S531L mutation). This technique employed modified primers with alterations at the 3′ end of the B2 primer, specifically designed for the target region using a manual approach, to ensure specificity for the mutated sequences. Although LAMP is highly sensitive, it may occasionally amplify products from the wild-type sequence. To enhance specificity for the S531L substitution, a single intentional mismatch was introduced into one primer of each primer set. Four LAMP primer sets were evaluated for their suitability using genomic DNA from both the wild-type *M. tuberculosis* H37Rv strain and the S531L mutant strain. Among these, only the primer set containing BIP and B2 was capable of detecting the mutation in this assay. Additionally, a second set of probes targeting the most common point mutations associated with rifampicin resistance in the *rpoB* gene of *M. tuberculosis* was also synthesized.

### 3.1. Optimum Temperature for the LAMP Reaction

**Figure 3:**
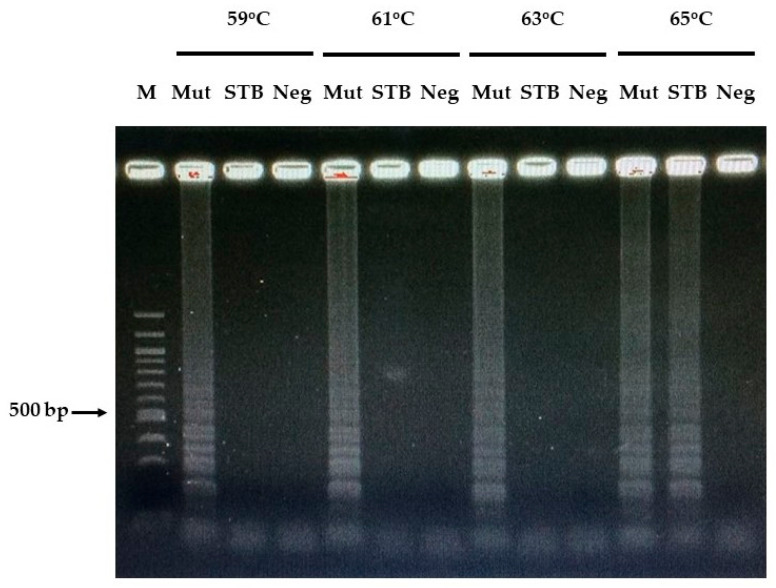
Optimal temperature for LAMP amplification. Lane 1, (M): ladder DNA marker; lanes 2–13: reaction for 60 min at 59, 61, 63, and 65 °C; Mut as MDR-TB; STB as H37RV; Neg as negative. All LAMP products were electrophoresed on 2% agarose gels.

We identified the optimal temperature range for the LAMP reaction to enable the rapid detection of *rpoB* gene mutations associated with rifampicin resistance in *M. tuberculosis.* Amplicons were observed after incubation at 59, 61, 63, and 65 °C; however, the most well-developed products were obtained at 59, 61, and 63 °C (Figure 3). The reaction efficiencies at these three temperatures were comparable. Therefore, the optimal temperature range for LAMP product detection was determined to be 59–63 °C, with a reaction time of 60 min required for detecting templates at low concentrations.

### 3.2. Optimum Concentration of MgSO_4_ for the LAMP Reaction

**Figure 4:**
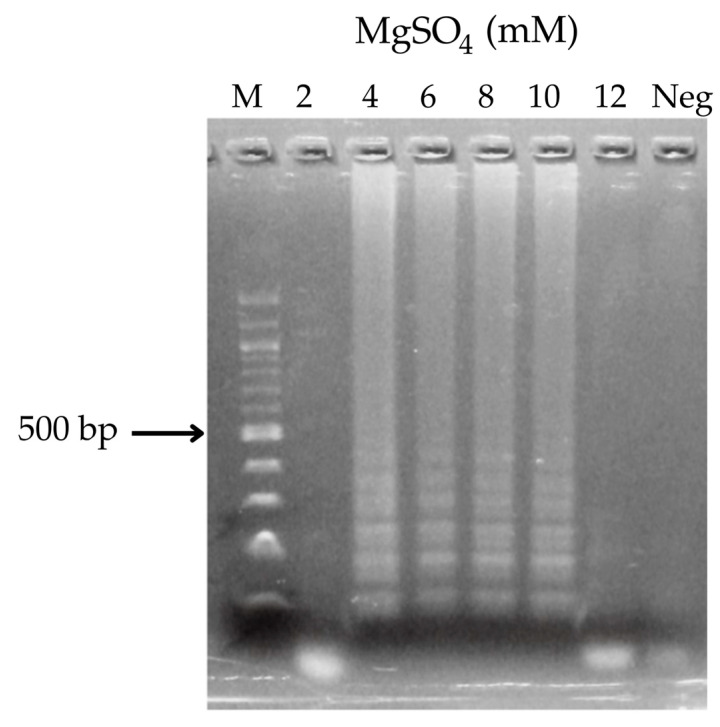
Optimal MgSO_4_ concentration for LAMP. Lane 1, M: ladder DNA marker; lanes 2–7: reaction for 60 min at 2, 4, 6, 8, 10, and 12 mM MgSO_4_; lane 8: negative control. All LAMP products were electrophoresed on 2% agarose gels.

The concentration of MgSO_4_ was optimized for the LAMP assay to detect specific *rpoB* gene mutations in *M. tuberculosis* associated with rifampicin resistance. Amplification products were observed at MgSO_4_ concentrations of 2, 4, 6, 8, 10, and 12 mM (Figure 4). The LAMP reaction was carried out using 6 mM of MgSO_4_ under the specified conditions. The resulting LAMP products were analyzed by 2% agarose gel electrophoresis.

### 3.3. dNTP Concentration in the LAMP Reaction

**Figure 5:**
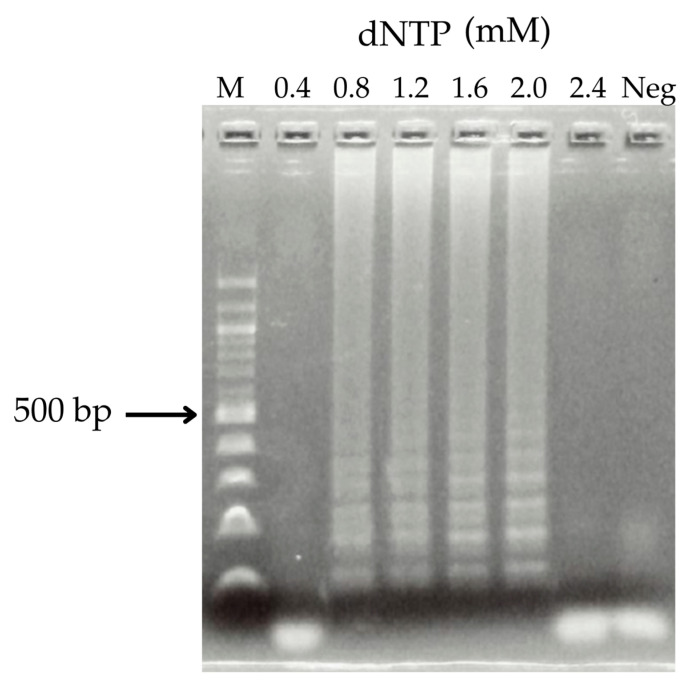
Optimal dNTP concentration for LAMP. Lane 1, M: ladder DNA marker; lanes 2–7: reaction for 60 min at 0.4, 0.8, 1.2, 1.6, 2.0, and 2.4 mM dNTP; lane 8: negative control. All LAMP products were electrophoresed on 2% agarose gels.

LAMP reactions were performed using tubes containing dNTP concentrations ranging from 5× to 10×. The optimal dNTP concentration for detecting *M. tuberculosis*-specific *rpoB* gene mutations associated with rifampicin resistance was determined to be 2.0 mM. Amplification products were observed at dNTP concentrations of 0.8, 1.2, 1.6, and 2.0 mM (Figure 5). The LAMP reaction was subsequently carried out using a dNTP concentration of 1.6 mM, under the same monitoring conditions as previously described. The resulting LAMP products were analyzed by 2% agarose gel electrophoresis.

### 3.4. Sensitivity and Specificity Test

**Figure 6:**
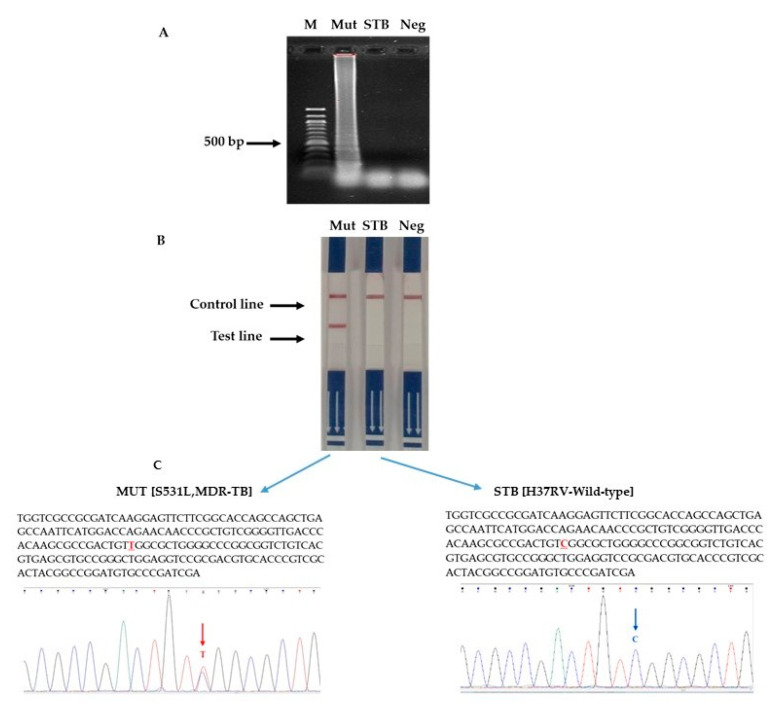
Specificity testing of the LAMP-LFD assay. (**A**) Lane 1, M: ladder DNA marker; lanes 2: Mut (MDR-TB), lanes 3: STB (standard strain H37RV), lanes 4: Neg (negative). (**B**) Corresponding results recorded visually via dipstick test. (**C**) DNA sequencing analysis.

**Figure 7:**
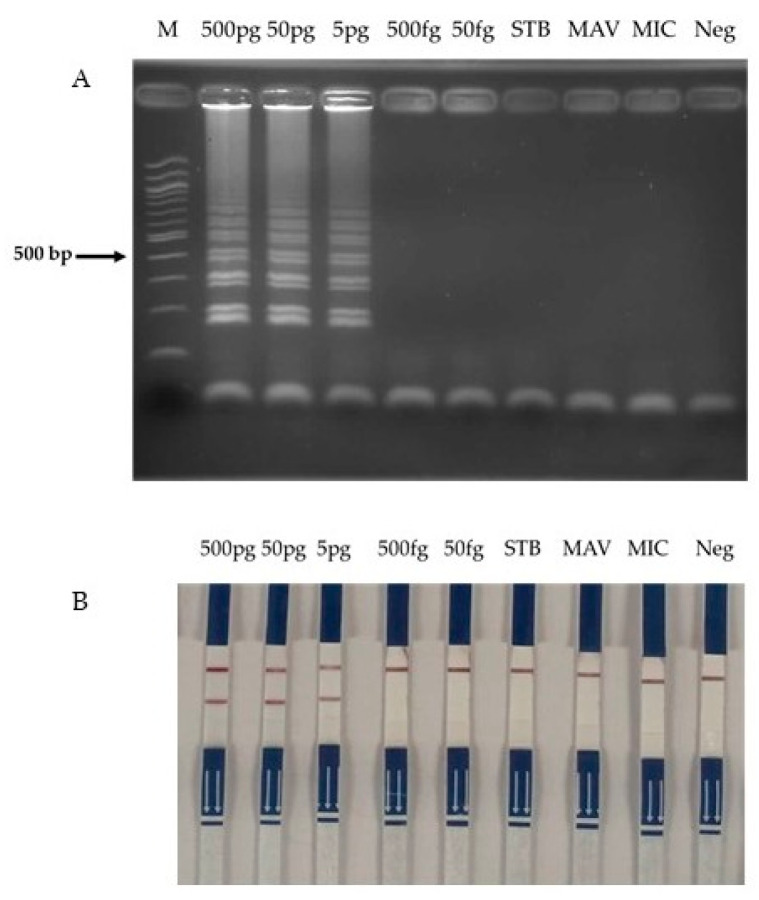
Detection sensitivity data of the LAMP-LFD assay for MDR-TB genomic DNA at concentrations ranging from 50 ng to 5 fg (initial concentration: 50 ng) were obtained from (**A**) LAMP and (**B**) LAMP-LFD assays. Lanes are designated as follows: M, DNA ladder marker; STB, standard strain H37Rv; MAV, *Mycobacterium avium*; MIC, *Mycobacterium intracellulare*; and Neg, negative control.

A specificity evaluation was performed using 50 ng of genomic DNA containing *rpoB* gene mutations associated with rifampicin resistance in *M. tuberculosis*. No cross-reactivity was detected across all the analytical platforms employed, including LAMP followed by agarose gel electrophoresis (Figure 6A), the LAMP-LFD assay (Figure 6B), and DNA sequencing (Figure 6C). In addition, the analytical sensitivity of the optimized LAMP-LFD assay was rigorously assessed through serial dilution experiments, revealing a lower detection limit of 5 pg of DNA, thereby demonstrating performance comparable to conventional drug susceptibility testing (DST) (Figure 7).

### 3.5. Performance of the LAMP-Combined Dipstick Assay for MDR-TB Diagnosis

All samples (*n* = 10) exhibited signatures of *rpoB* gene mutations associated with rifampicin resistance in *M. stuberculosis*. A subsequent investigation assessed whether the resistant strains carried polymorphisms at codon 94 of the *rpoB* gene. The dipstick assay results were compared with those of drug susceptibility testing (DST) (Table 2). The dipstick test correctly identified all six mutations in the six resistant samples, showing complete concordance with both DST and DNA sequencing, with a sensitivity of 100% [6/6 samples].

## 4. Discussion

Based on the molecular properties of MDR-TB and the significant mutations associated with rifampicin resistance in the *rpoB* gene of *M. tuberculosis*, we developed a two-tier detection algorithm. The strategy employed the LAMP method, targeting a 96 base pair region of the *rpoB* gene, and was combined with hybridization and detection using an LFD assay (Figure 6B). The areas of the *rpoB* gene harboring MDR-TB-associated mutations were focused on with mutation-specific probe sets for diagnosing resistance-determining substitutions associated with rifampicin resistance. It can be separated and gives results in the same direction as previous studies [20,23].

LAMP is an emerging nucleic acid amplification technique, which is simple and highly sensitive for the early diagnosis of target gene DNA. Its isothermal nature, high specificity, and short turnaround time render it a feasible alternative to traditional PCR, especially for field assays [13,14]. In the current study, we developed LAMP primers to differentiate a single-point mutation. The primer B2 was selectively adapted to detect only the mutant sequences. Accordingly, PCR products were amplified only in the presence of the mutant allele and not with the wild-type genomic DNA [20].

Primer annealing was targeted to the first or third positions from the 3′ end of the B2 primer to detect the S531L mutation (C→T, C→G). To achieve complete coverage of all base substitutions, a degenerate base “M” was used in the first position with the introduction of the specific mismatch base “W” at the third position to increase mutation specificity. Moreover, TTTT spacers were added in the F1c-F2 and B1c-B2 intervals in the FIP and BIP primers, respectively, to enhance primer template annealing. This assay design may also be extended to the detection of other clinically relevant SNPs in different pathogens [20,23]. In this study, the primer set was designed as shown in Table 1 to detect rifampicin resistance in the *rpoB* gene of *M. tuberculosis*. The LAMP-LFD assay was developed and evaluated using clinical samples suspected of drug resistance. The designed primer set was specific to the S531L mutation (C→T), and the assay was tested for both specificity and sensitivity. It successfully distinguished rifampicin-resistant strains harboring the *rpoB* mutation from wild-type *M. tuberculosis*. The assay results were confirmed through DNA sequencing, which revealed that all tested samples carried the S531L mutation (C→T), indicating the primer’s high specificity to this mutation. However, upon further investigation, it was found that the S531L mutation may also occur due to a nucleotide change from C→G. To enable future detection of this variant, the researchers plan to develop and redesign the primer set to specifically recognize the S531L mutation (C→G) as well, thereby covering all possible base changes associated with this mutation. In this regard, degenerate base design will be employed, wherein the degenerate base “M” will be used at the first position, along with the incorporation of a specific mismatch base “W” at the third position to enhance mutation specificity. This future primer development aims to ensure the broader detection of the S531L mutation regardless of the nucleotide substitution involved.

Thus, most sensitive nucleic acid assays depend on sophisticated instruments and toxicants, such as ethidium bromide, for visualization. By comparison, the LAMP amplicons can be visualized by magnesium pyrophosphate precipitation (visible as white turbidity), dye detection, and turbidity measurements. Nevertheless, these methods still do not appear to be highly specific in detecting target amplicons. To minimize this problem, cationic polymer precipitation and sequence-specific hybridization probes, such as rifampicin-labeled probes, can be employed for detection. However, they are expensive and also take a long time to process; therefore, field deployment is impossible [9].

The LFD assay can provide a convenient, easy, and cost-effective alternative method, offering a visual readout compared to the hybridization assay and membrane chromatography. The results from LAMP can be read in 10–15 min once LAMP is complete with a simple heat source, compared to gel electrophoresis, which requires 45–60 min. Additionally, the operation of dipsticks does not require an expensive instrument, and thus they are adaptable to resource-limited conditions. However, the sensitivity is not high enough to detect low-producing nucleic acids [25,26,27]. Hence, they are best suited to amplification techniques like LAMP. For instance, GenoQuick has combined PCR with a strip assay for the clinical detection of *M. tuberculosis*, and the feasibility of combining LAMP-LFD has been confirmed in previous reports.

Herein, we established a LAMP-LFD assay to detect rifampicin-resistant *M. tuberculosis* carrying the S531L [*rpoB*, C→T/G] mutation sensitively and straightforwardly. The optimum reaction temperature was 59–63 °C, and the total reaction time was 60 min. Magnesium sulfate and dNTP concentrations were determined for maximal reaction efficiency, as described previously. The appropriate concentration range of MgSO_4_ was determined to be 4–10 mM, with concentrations above 10 mM exhibiting a moderately inhibitory effect on enzyme activity. A concentration of 6 mM was selected as it was sufficiently high to support amplification, despite the minimum effective concentration being 4 mM when using a DNA template at 5 ng. However, 4 mM of MgSO_4_ could compromise the discrimination of SNPs at low DNA concentrations. Therefore, 6 mM was considered more appropriate, providing an optimal balance for differentiating between rifampicin-resistant strains carrying *rpoB* gene mutations and wild-type *M. tuberculosis*.

The optimal concentration of dNTPs was established by evaluating LAMP reactions across a gradient of increasing dNTP concentrations to identify the most effective condition. LAMP product formation was observed within the range of 0.8–2.0 mM. A concentration of 1.6 mM was chosen for subsequent experiments, as it adequately supported the amplification process without being so low as to result in insufficient product formation. Moreover, this concentration was suitable for discriminating between rifampicin-resistant *rpoB* mutants and wild-type *M. tuberculosis*. Conversely, 2.0 mM was not selected because excessively high dNTP concentrations can increase the risk of false positive results or inhibit the amplification reaction, as evidenced by the absence of amplification at 2.4 mM. Thus, 1.6 mM was deemed optimal, balancing reaction efficiency with cost-effectiveness by avoiding unnecessary reagent use. The optimal conditions were 6 mM of MgSO_4_, 1.6 mM of dNTPs, and 8 U of *Bst* DNA polymerase, which enabled the reliable detection of mutations.

Superior primers were well-designed based on the C→T and C→G changes in codon 531 of the *rpoB* gene, which encodes the β-subunit of RNA polymerase. This protein is the key to transcription and is the primary target of rifampicin, where it binds. The S531L mutation is essentially detrimental for binding to rifampicin; its occurrence is associated with the development of resistant strains.

For assay specificity, the 3′ terminus of the B2 primer was manually adjusted using primer design software (https://primerexplorer.eiken.co.jp/lampv5e/index.html (accessed on 21 August 2025)). Although the LAMP technique is particular, it is sensitive to the amplification of non-target sequences. To inhibit this, a deliberate mismatch was also engineered to increase the specificity of the S531L mutation and minimize the chances of false positives.

Four sets of LAMP primers were tested with gDNA from wild-type H37Rv and *M. tuberculosis* S531L mutant strains. Of these, the combination of BIP and B2 could specifically identify the mutant strain. This result also highlights the importance of optimal primer design in molecular diagnostics.

While LAMP represents a promising approach for SNP detection, its application is accompanied by several methodological challenges, particularly in primer design. The technique necessitates the use of allele-specific primers tailored to each SNP, which must be meticulously designed to ensure high specificity and minimize false positive signals. Inadequate primer design may result in nonspecific amplification products or the formation of primer dimers, especially during the later phases of the amplification reaction [20].

Moreover, the output of LAMP assays is typically semi-quantitative, providing a binary readout indicative of the presence or absence of the target sequence, rather than offering precise quantitative data comparable to that obtained from quantitative polymerase chain reactions (qPCRs). Consequently, LAMP is most effective for the targeted detection of known, well-characterized SNPs, but it is less suitable for the simultaneous identification of multiple or novel variants. In such contexts, high-throughput genotyping platforms such as next-generation sequencing (NGS) offer superior analytical resolution and multiplexing capability.

A second set of primers can be developed to address other common mutations in the *rpoB* gene conferring rifampin resistance. This strategic extension highlights the general public health threat of reliable diagnostics and the importance of tracking the emergence of resistance in the future. The inclusion of tools such as LAMP in the diagnostic chain provides clinicians and researchers with immediate access to detect and counteract hardy TB strains.

This study evaluated only ten actual clinical samples, as it served as a preliminary investigation aimed at demonstrating the capability to differentiate SNPs through primer design similar to that studied previously [20]. Therefore, the primary focus of this research was on the design of primers. Nevertheless, the research team intends to conduct further evaluations involving a larger sample size to assess the qualitative performance of the LAMP-LFD method in the future.

This novel LAMP-LFD method enables the detection of SNPs in the *rpoB* gene at codon 531, a mutation commonly associated with rifampicin resistance in *M. tuberculosis*. It represents a promising first-line diagnostic tool for the rapid and accurate identification of drug-resistant TB cases. The assay offers significant advantages, including a straightforward visual readout, minimal technical requirements, low cost, and ease of implementation in field settings. These attributes make it particularly well-suited for use in resource-limited environments. Importantly, the method facilitates early diagnosis and timely clinical management, thereby contributing to reduced disease transmission and lowering both the direct and indirect costs associated with tuberculosis monitoring and treatment.

## 5. Conclusions

The combination of LAMP with dipstick technology provides a robust, user-friendly, and sensitive assay for the rapid detection of clinically relevant mutations, such as S531L. This system is particularly fit for use in resource-poor settings where more complex diagnostics are less feasible. Continued advances in molecular diagnostics offer considerable potential to improve patient care and address the growing crisis of antimicrobial resistance.

## Figures and Tables

**Table 1:** Primers and probe used for LAMP of the S531L *rpoB* gene mutation and rifampicin drug resistance in *M. tuberculosis*.

Primer	Sequence
F3	5′-CCGCAGACGTTGATCAACAT-3′
B3	5′-CCCCTCAGGGGTTTCGA-3′
FIP	5′-GTGCCGAAGAACTCCTTGATTTTTCGGCCGGTGGTCGCCG-3′
BIP-bio	5′-biotin-CAACCCGCTGTCGGGGTTTTTTGCCGGGCCCCAGCGTCA-3′
LB-fit-C	5′-fitC-ACCGACAAGCGCCGACTG-3′

**Table 2:** Detection of MDR-TB members via the dipstick assay. Results obtained by the dipstick assay were compared to those obtained via the DST and DNA sequencing assay.

DST */DNA Sequencing	LAMP Dipstick		
	Positive	Negative	Total
*rpoB* resistance (6)	6	0	6
*rpoB* susceptible (4)	0	4	4
Total (10)	6	4	10

* The test results were obtained from the National Tuberculosis Reference and Diagnostic Laboratory, Department of Disease Control, Ministry of Public Health, Thailand.

## Data Availability

The datasets used in the present study are available from the corresponding author upon reasonable request.
